# MRI evaluation of clinical remission in juvenile idiopathic arthritis

**DOI:** 10.1186/1546-0096-9-S1-P114

**Published:** 2011-09-14

**Authors:** M van Veenendaal, R Hemke, MI Bos, M Maas, MAJ van Rossum, TW Kuijpers

**Affiliations:** 1 Department of Pediatric Rheumatology, Emma Children’s Hospital Academic Medical Center, Amsterdam, The Netherlands; 2 Department of Radiology, Academic Medical Center, Amsterdam, The Netherlands; 3 Department of Pediatric Rheumatology, Jan van Breemen Institute, Amsterdam, The Netherlands

## Background

Despite clinical remission a substantial proportion of Juvenile Idiopathic Arthritis (JIA) patients will flare after a period of inactive disease, suggesting ongoing disease activity which cannot be detected by current assessment methods. As MRI has proven to depict subclinical inflammation in JIA, it may identify patients at risk for flaring.

## Aim

The purpose of this study was to use MRI to evaluate the knee for signs of subclinical inflammation in JIA patients who are in clinical remission.

## Methods

Prospective cohort study of 30 patients with JIA (median age 13.5 years [IQR,10.6-15.8], median disease duration 3.9 years [IQR,2.0-4.0]) and fulfilling the PRINTO remission criteria. Contrast-enhanced MRI of the previously most involved knee was used to evaluate the degree of synovial hypertrophy (rated: absent, < 3 mm or > 3 mm synovial thickening).

## Results

Data of 30 patients were analysed: 26 patients in clinical remission on medication (CRM) (median duration inactive disease 0.9 years [IQ,0.6-1.2]) and 4 patients who achieved clinical remission off medication (CR) (median duration inactive disease 2.4 years [IQR,1.5-3.1]). Thirty percent of CRM patients and 1 of 4 CR patients had > 3 mm synovial thickening, suggestive for active synovitis.

## Conclusion

Numerous JIA patients who satisfy the PRINTO remission criteria had MRI detected synovitis in this study. Subclinical inflammation may be the reason for the high percentage of JIA patients who flare after tapering medication. MRI may be a sensitive tool to define true remission and may identify JIA patients at risk for flaring of disease.

